# Impact of preoperative education on early outcomes following hip and knee arthroplasty: a 21,000-patient multicentre NHS analysis

**DOI:** 10.1302/2633-1462.74.BJO-2025-0259.R1

**Published:** 2026-04-21

**Authors:** Kashif I. Ahmad, Antonio Eleuteri, Tom Jenks, Phillipa Thorpe

**Affiliations:** 1 NHS University Hospitals of Liverpool Group, Liverpool, UK; 2 Department of Physics, School of Physical Sciences, University of Liverpool, Liverpool, UK; 3 School of Medical Sciences, Faculty of Biology, Medicine, and Health, University of Manchester, Manchester, UK; 4 Advancing Quality Alliance (AQuA), Manchester, UK

**Keywords:** Preoperative education, Arthroplasty, Length of stay, Early mobilization, Readmission rates, hip and knee arthroplasty, hip and knee arthroplasty, frailty, comorbidity, Hip, arthroplasty, total knee arthroplasty (TKA), postoperative complications, end-stage osteoarthritis, elective arthroplasty

## Abstract

**Aims:**

Preoperative education is routinely integrated into enhanced recovery pathways for hip and knee arthroplasty, yet its independent effect on early outcomes remains uncertain. This study examined whether completion of structured preoperative education was associated with length of stay (LOS), early mobilization, and 30-day readmission, adjusting for patient risk factors including frailty.

**Methods:**

A retrospective study was performed using routinely collected data from adults undergoing elective primary hip or knee arthroplasty across 17 NHS and independent hospitals (2019 to 2024). Completion of preoperative education (Advancing Quality measure HK-07) was the primary exposure. LOS and 30-day readmission were primary outcomes; early mobilization within 24 hours (HK-05) was secondary. Multivariable regression models were adjusted for age, sex, procedure type, comorbidity category, and Hospital Frailty Risk Score, with restricted cubic splines for non-linear age effects and cluster-robust estimation for site variation.

**Results:**

Of 21,225 included patients, 93.4% completed preoperative education and 97.2% mobilized within 24 hours. Completion of education was associated with a modest reduction in LOS, with the greatest effect observed in younger patients and attenuation beyond 70 years of age. No significant associations were observed between education and 30-day readmission, whereas completion of education was associated with early mobilization. Increasing frailty and comorbidity were associated with longer LOS and higher readmission risk.

**Conclusion:**

Preoperative education is associated with a shorter hospital stay for younger and lower-risk patients but does not appear to influence 30-day readmission. Frailty and comorbidity remain dominant drivers of outcome variation. Education should be considered one component of perioperative care, and additional tailored support may be required for older and frailer individuals.

Cite this article: *Bone Jt Open* 2026;7(4):566–573.

## Introduction

Hip and knee arthroplasty are highly effective interventions for end-stage osteoarthritis, delivering substantial improvements in pain, mobility, and quality of life.^1,2^ To meet rising demand, hospitals across the UK have widely adopted enhanced recovery pathways, which emphasize patient education, early mobilization, and streamlined discharge processes.^2-4^ Among these, preoperative education is intended to prepare patients for surgery, optimize expectations, and encourage engagement with recovery activities, improving understanding, confidence, and adherence to postoperative care. However, evidence supporting its impact on early outcomes remains inconsistent.^5,6^

Evidence evaluating the effect of preoperative education in routine practice is limited. Most studies have been conducted in single centres, whereas delivery across the NHS varies in content, format, and timing.^3,4,7,8^ As elective arthroplasty volumes continue to grow, understanding whether education is associated with postoperative length of stay (LOS), mobilization, and readmission is increasingly relevant to service planning.^9-11^ Clarifying where education delivers most value may support optimization of elective pathways and resource allocation across NHS providers.

Patient factors such as age, comorbidity, and frailty also contribute strongly to perioperative risk and variation in outcomes.^9,12^ Frailty is now recognized as an important determinant of recovery following arthroplasty and has been associated with prolonged hospitalization, postoperative complications, and reattendance.^5,6,9-17^ However, it is unclear to what extent frail patients derive the same benefit from standardized preoperative education programmes as healthier individuals, and frailty may introduce barriers to accessing or applying information provided in standard sessions.

This study aimed to evaluate whether completion of preoperative education is associated with LOS, early mobilization, and 30-day readmission following hip and knee arthroplasty, adjusting for frailty and other patient risk factors.

## Methods

### Study design and setting

We conducted a retrospective study using routinely collected data from patients undergoing primary hip or knee arthroplasty across 17 NHS and independent hospitals in the North West of England between January 2019 and December 2024. Data were obtained from the Advancing Quality (AQ) Elective Hip and Knee Replacement Programme, an ongoing regional initiative that prospectively records perioperative process measures and outcomes to benchmark and improve care across providers.^8^ As this study analyzed the complete population captured during the study period, no a priori power calculation was required.

### Participants and eligibility criteria

**Fig. 1:**
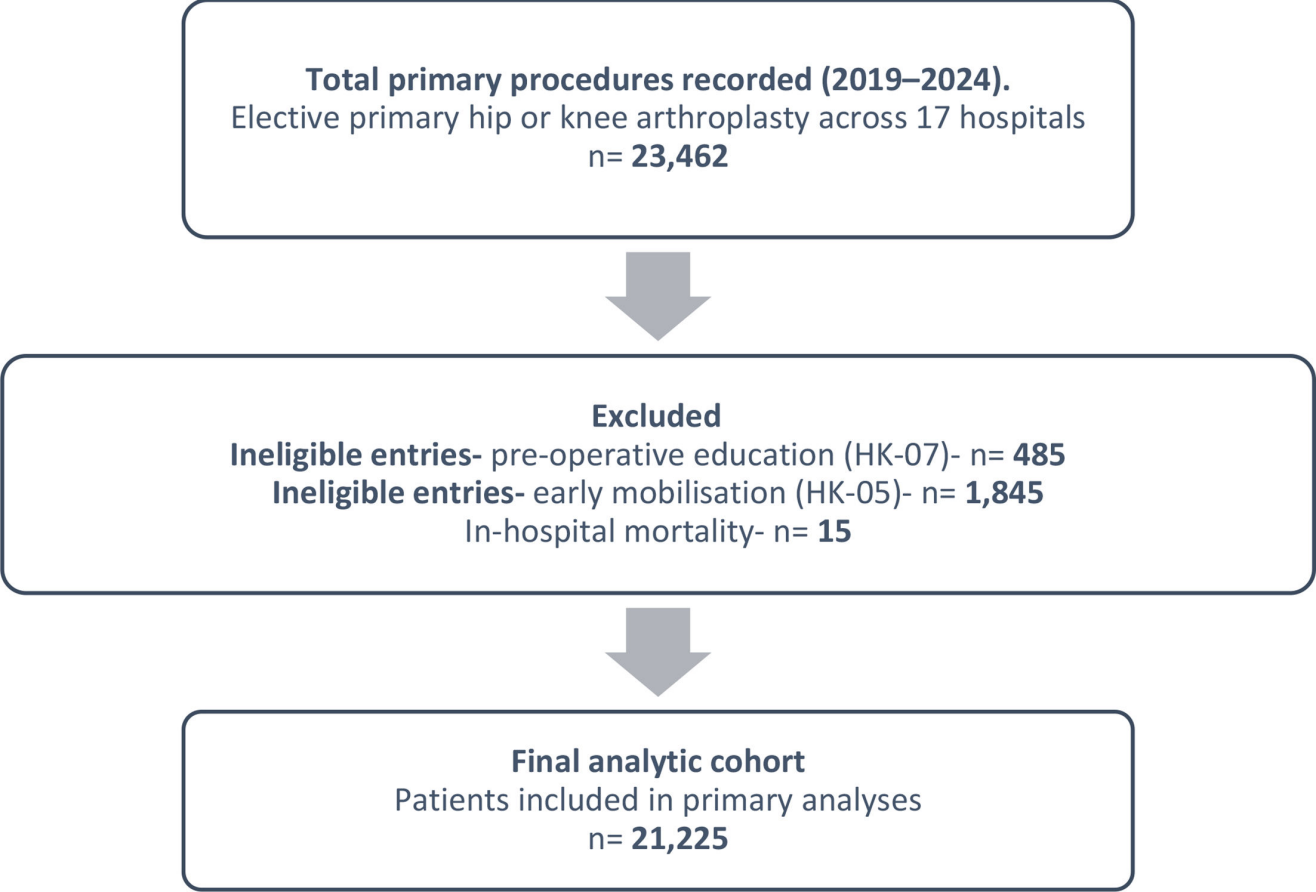
Flow diagram of cohort selection and exclusions. Exclusions from individual process measures reflect patient refusal or clinical contraindications.

All adults undergoing elective primary total hip arthroplasty (THA) or total knee arthroplasty (TKA) were eligible for inclusion. Exclusion criteria comprised revision arthroplasty, non-elective procedures, patients recorded as exempt from both preoperative education and mobilization measures, and in-hospital mortality (excluded from readmission analysis). The initial dataset included 23,462 procedures. After exclusions for ineligible entries on preoperative education (n = 485) and early mobilization (n = 1,845), reflecting patient refusal or clinical contraindications, and excluding in-hospital mortality (n = 15), a final cohort of 21,225 patients was available for analysis (Figure 1).

### Participant characteristics

Among included patients, 10,098 (47.7%) underwent THA and 11,069 (52.3%) TKA. The median age was 70 years (IQR 62 to 76), with 43.4% male and 56.6% female. Preoperative education (HK-07) was completed by 93.4% of patients, and 97.2% achieved early mobilization within 24 hours (HK-05). The median LOS was three days (IQR 2 to 4), and 30-day readmission occurred in 6.4% of patients (Table I). Completion of AQ measures varied across sites, with marked interorganizational heterogeneity illustrated in subsequent tables and figures.

**Table I.** Descriptive statistics.

Variable	Value	Missing, n
Hip/knee, n (%)	Hip: 10,098 (47.7); Knee: 11,069 (52.3)	58
Trust, n (%)	Blackpool: 750 (3.5); Countess of Chester: 376 (1.8); East Lancs: 559 (2.6); Euxton Hall: 1,477 (7.0); Fairfield Independent: 1,118 (5.3); Fulwood Hall: 1,562 (7.4); Lancs Teaching: 1,031 (4.9); Liverpool University Hospitals: 2,769 (13); Manchester FT: 2,942 (14); Mersey & W Lancs: 606 (2.9); Mid Cheshire: 1,258 (5.9); Northern Care Alliance: 2,000 (9.4); Renacres: 1,525 (7.2); Spire Cheshire: 480 (2.3); Spire Liverpool: 474 (2.2); Warrington & Halton: 510 (2.4); WWL: 1,788 (8.4)	0
Median LOS, days (IQR)	3 (2 to 4)	0
Median age, yrs (IQR)	70 (62 to 76)	0
Sex, n (%)	Male: 9,212 (43.4); Female: 12,004 (56.6)	9
Readmission 30 days, n (%)	Yes: 1,354 (6.4); No: 19,871 (93.6)	0
Early mobilization, n (%)	Pass: 20,639 (97.2); Fail: 586 (2.8)	0
Preoperative education, n (%)	Pass: 19,818 (93.4); Fail: 1,407 (6.6)	0
Charlson (grouped), n (%)	None: 9,947 (46.9); Low: 7,540 (35.5); Moderate: 3,738 (17.6)	0

LOS, length of stay; WWL, Wrightington, Wigan and Leigh Teaching Hospitals NHS Foundation Trust.

### Exposure and process measures

The primary exposure variable was completion of preoperative education, as defined by AQ process measure HK-07. Completion was recorded when patients attended, engaged with, or received the structured preoperative information session as determined by local site implementation. Education content varied between providers and was determined locally; commonly reported components included information on the surgical procedure, pain control, mobilization expectations, and discharge planning. Delivery was either in-person, group-based, one-to-one, or digital depending on local resources. Non-completion reflected non-attendance, withdrawal, or clinician-documented inability to engage.

Early postoperative mobilization was defined by AQ process measure HK-05 as achievement of walking within 24 hours of surgery, as recorded by the clinical team according to local practice. For both HK-05 and HK-07, outcomes were recorded as pass or fail.

### Outcome measures

Primary outcomes were hospital LOS, measured in days from admission to discharge among patients alive at hospital discharge, and emergency, unplanned readmission within 30 days of hospital discharge, excluding planned elective admissions. Early mobilization within 24 hours (pass/ fail) was assessed as a secondary outcome.

### Covariates

Demographic and clinical covariates included age, sex, procedure type (THA/ TKA), and Charlson Comorbidity Index (CCI),^18^ grouped into three categories (0, 1 to 2, and ≥ 3). Frailty was assessed using the Hospital Frailty Risk Score (HFRS), categorized as none (0), low,^1,2^ or intermediate/high (≥ 3). Although originally developed in older adults, the HFRS is now widely used as a perioperative risk stratification tool across adult surgical cohorts and forms part of routine AQ reporting.^11-17^

### Ethics and data governance

As this study analyzed routinely collected anonymized service-evaluation data, it did not require NHS Research Ethics Committee approval in accordance with Health Research Authority guidance. The AQ dataset is held under regional data-sharing agreements between participating organizations, and data access for this work was approved through the programme’s governance framework.

### Statistical analysis

Descriptive analyses summarized cohort characteristics using medians and IQRs for continuous variables and counts and percentages for categorical variables. LOS was right-skewed; therefore, a Box-Cox transformation was applied, with the optimal parameter (*a* = 0.415) selected by maximization of the profile likelihood.^19,20^ To account for potential non-linear effects, age was modelled using restricted cubic splines based on established regression modelling principles.^19,20^ Linear regression assessed associations of education completion and early mobilization with LOS. Logistic regression assessed associations of education completion and early mobilization with 30-day readmission. Both models adjusted for age, sex, procedure type, CCI category, and frailty category. Model effects were assessed using χ² statistics with corresponding p-values. Furthermore, logistic regression assessed the association of education completion with early mobilization. Site-level clustering was addressed using robust estimators of the standard errors of the parameters of all the models. A family-wise significance level of 0.05 was applied across the three outcomes using a Bonferroni adjustment, giving an effective threshold of approximately 0.02 per outcome.^19^ Model calibration and internal validation were examined using bootstrap resampling.^19^ Missing values for sex and mobilization (< 0.5% of entries) were replaced with the most frequent category within the relevant field;^19^ no multiple imputation was undertaken. Analyses were performed in R package (R Foundation for Statistical Computing, Austria).

## Results

### Cohort and exclusions

A total of 23,462 patients underwent elective primary hip or knee arthroplasty during the study period across 17 NHS and independent hospitals. After excluding patients with ineligible entries for preoperative education (n = 485) and early mobilization (n = 1,845), reflecting patient refusal or clinical contraindications, and excluding in-hospital mortality (n = 15), 21,225 patients remained for analysis (Figure 1). This represents 90.5% of the total submitted dataset.

### Primary outcome: length of stay

**Fig. 2:**
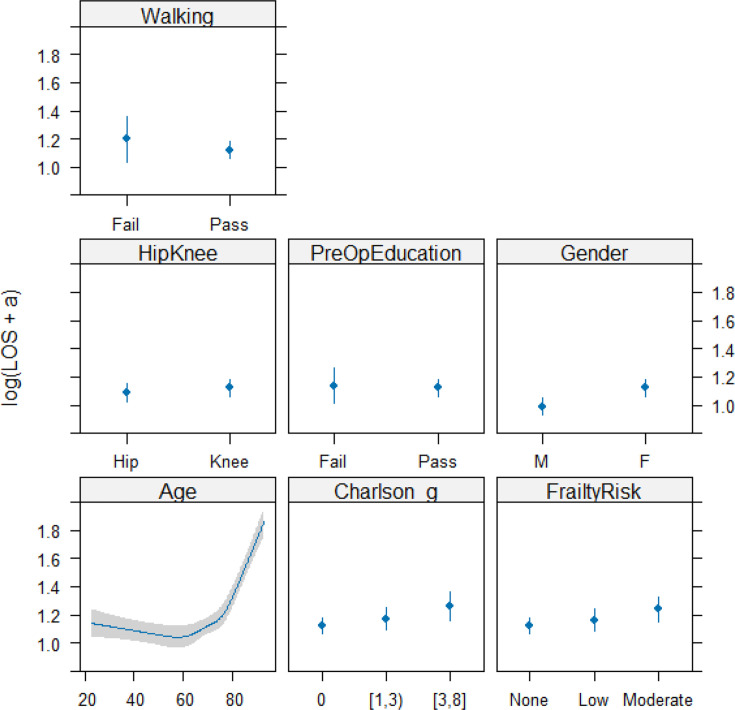
Predicted length of stay (LOS) with a 95% confidence limit on effects.

**Fig. 3:**
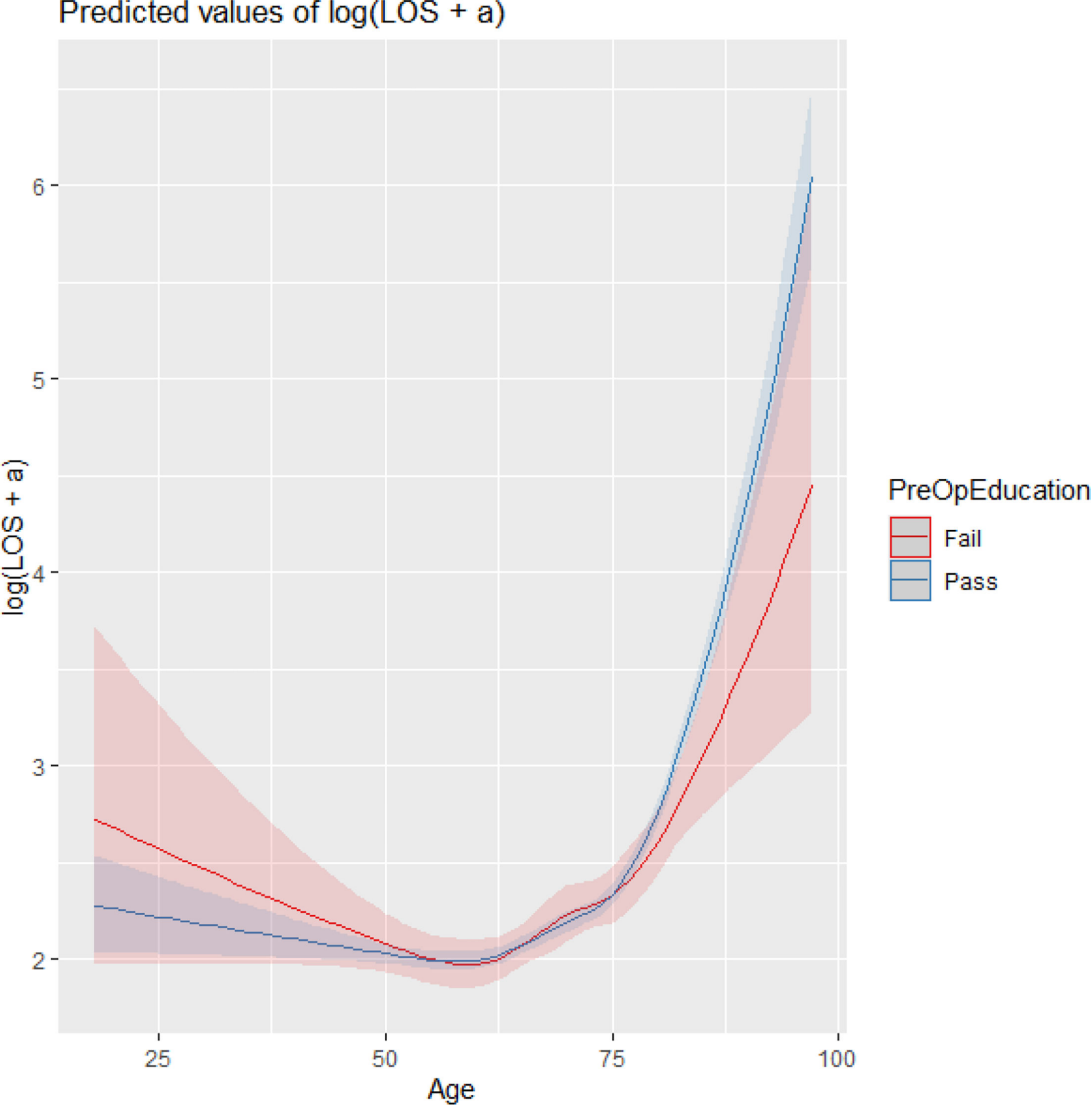
Predicted length of stay (LOS) by age and preoperative education with a 95% confidence limit on effects.

Completion of preoperative education was associated with a modest but statistically significant reduction in LOS after adjustment for age, comorbidity, and frailty (Table II). The magnitude of benefit differed by age, with the strongest effect observed in patients aged under approximately 50 years (Figure 2 and Figure 3). In this sub-group, predicted LOS was approximately 0.5 to 0.8 days shorter on average among those completing education compared with those who did not. Beyond age ~ 70 years, the difference attenuated and no meaningful advantage was observed.

**Table II.** *F* statistics of length of stay model with effect sizes and a 95% CI.

Factor	*F* (df,21208)	df	p-value
Hip/Knee	8.8	1	0.003
Age	107	8	< 0.001
Non-linear	137	6	< 0.001
Age, preoperative education interaction*	6.3	4	< 0.001
Preoperative education	5.1	5	< 0.001
Sex	107	1	< 0.001
Walking	1.2	1	0.300
Frailty risk	10	2	< 0.001
Charlson grouped	9.3	2	< 0.001

* Sub analysis within age- preop education interaction.

df, degrees of freedom.

**Fig. 4:**
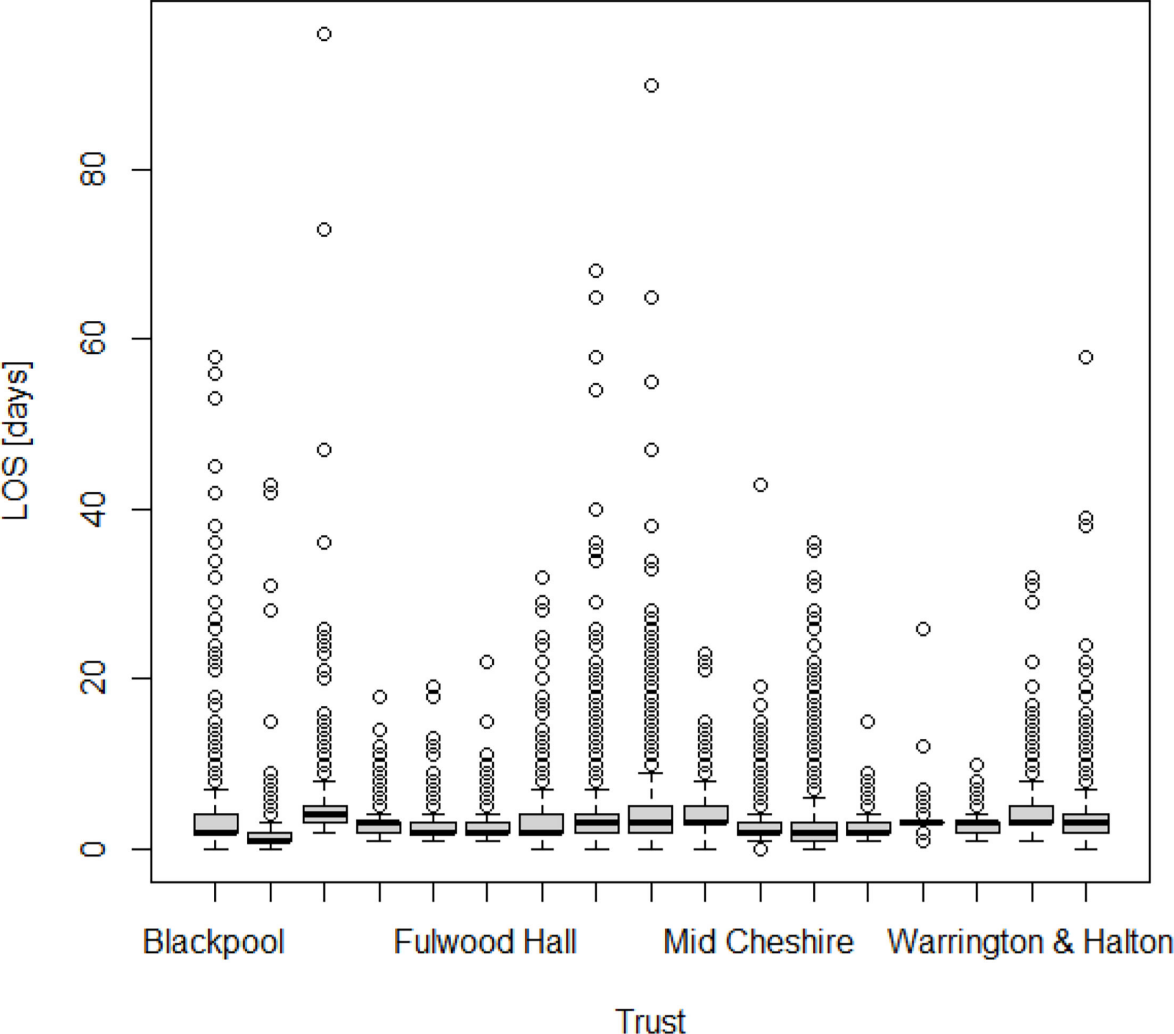
Boxplots of length of stay (LOS) by trust.

**Fig. 5:**
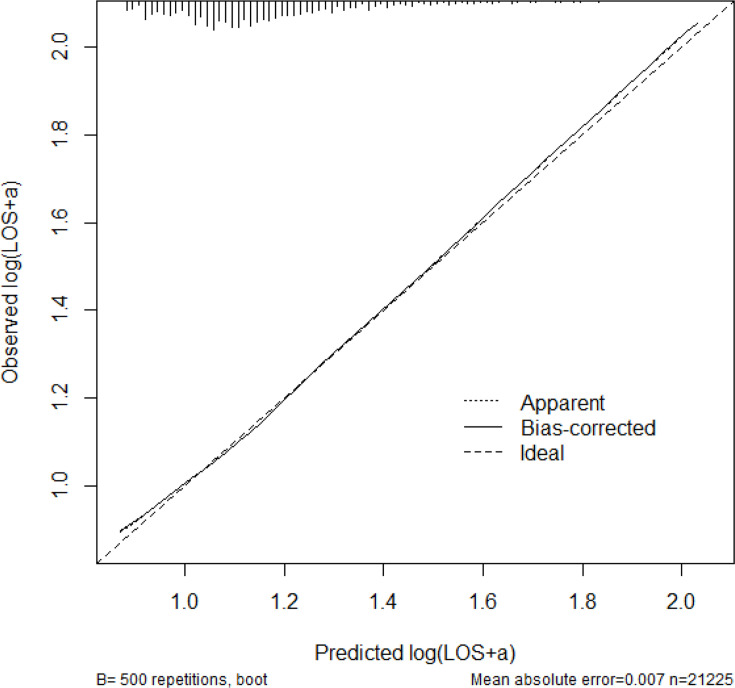
Calibration of length of stay (LOS) prediction.

LOS increased progressively with rising frailty category and CCI score. Compared with patients with no frailty, those with intermediate/high frailty had substantially longer predicted LOS. Knee arthroplasty and female sex were also associated with increased LOS. The distribution of LOS across trusts is visualized in Figure 4, outlining the necessity of both the Box-Cox transformation and site-level clustering correction of the statistics. A model calibration plot (Figure 5) demonstrates acceptable internal validity.^19^

### Primary outcome: 30-day readmission

**Fig. 6:**
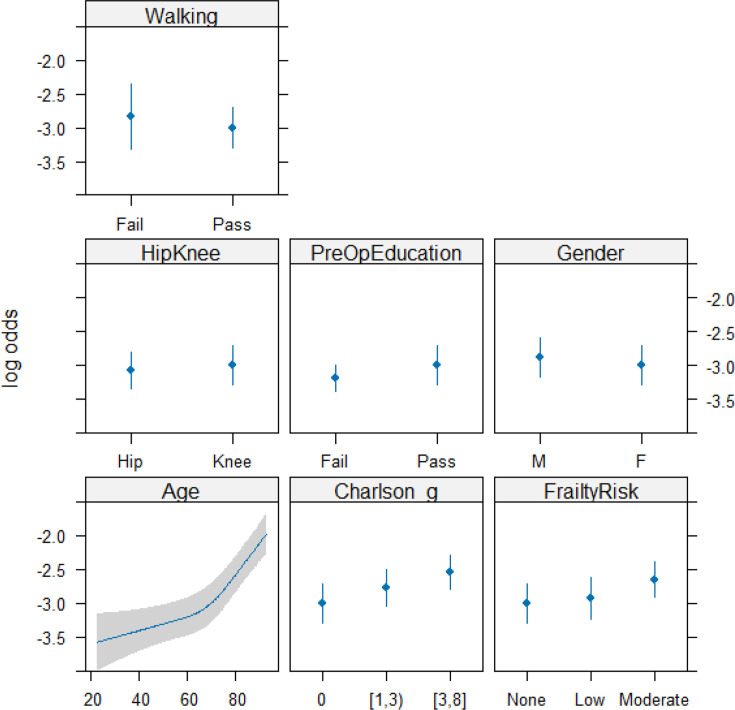
Predicted log-odds of readmission with a 95% confidence limit on effects.

**Fig. 7:**
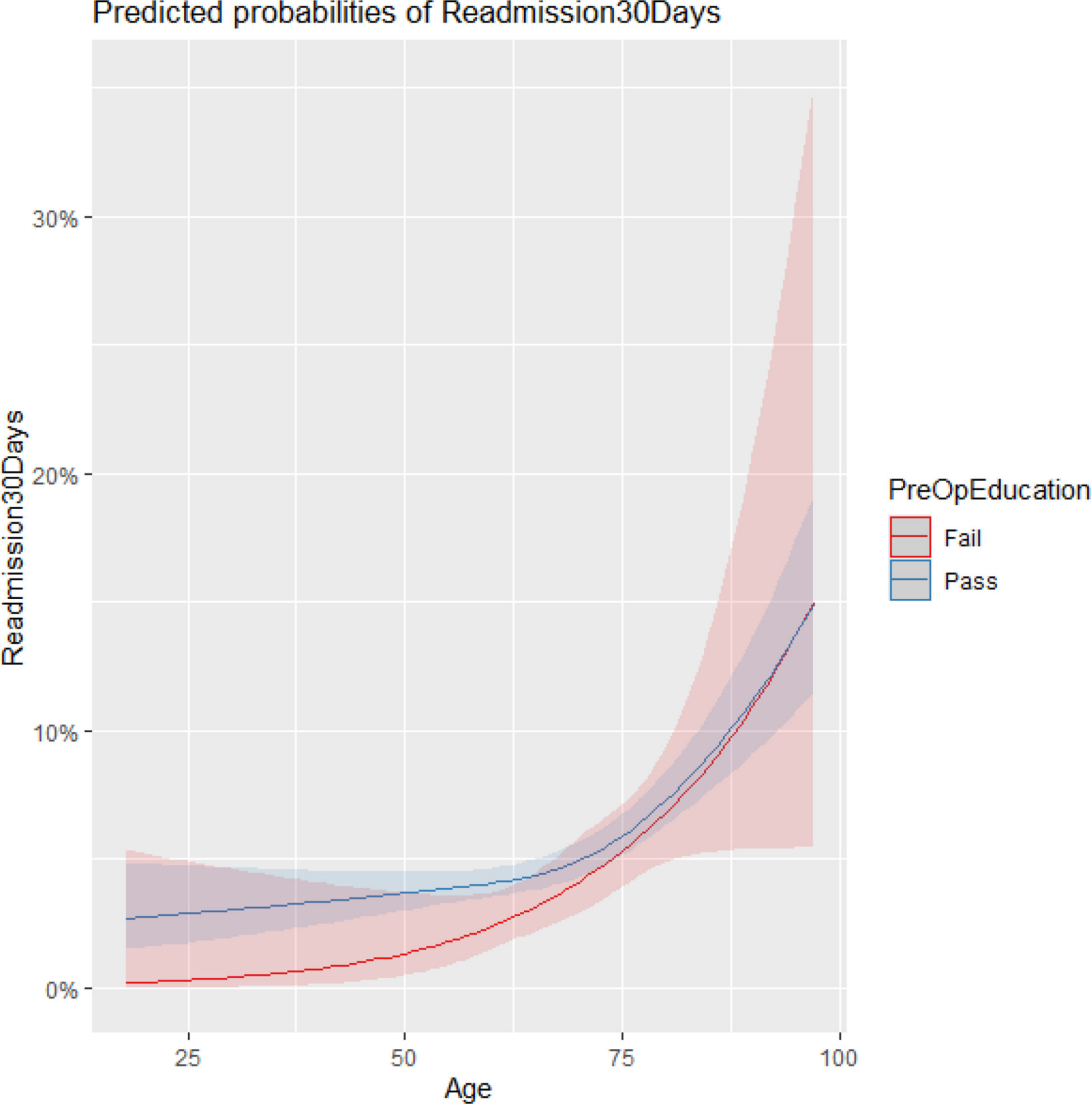
Predicted probability of readmission by age and preop education with a 95% confidence limit.

**Fig. 8:**
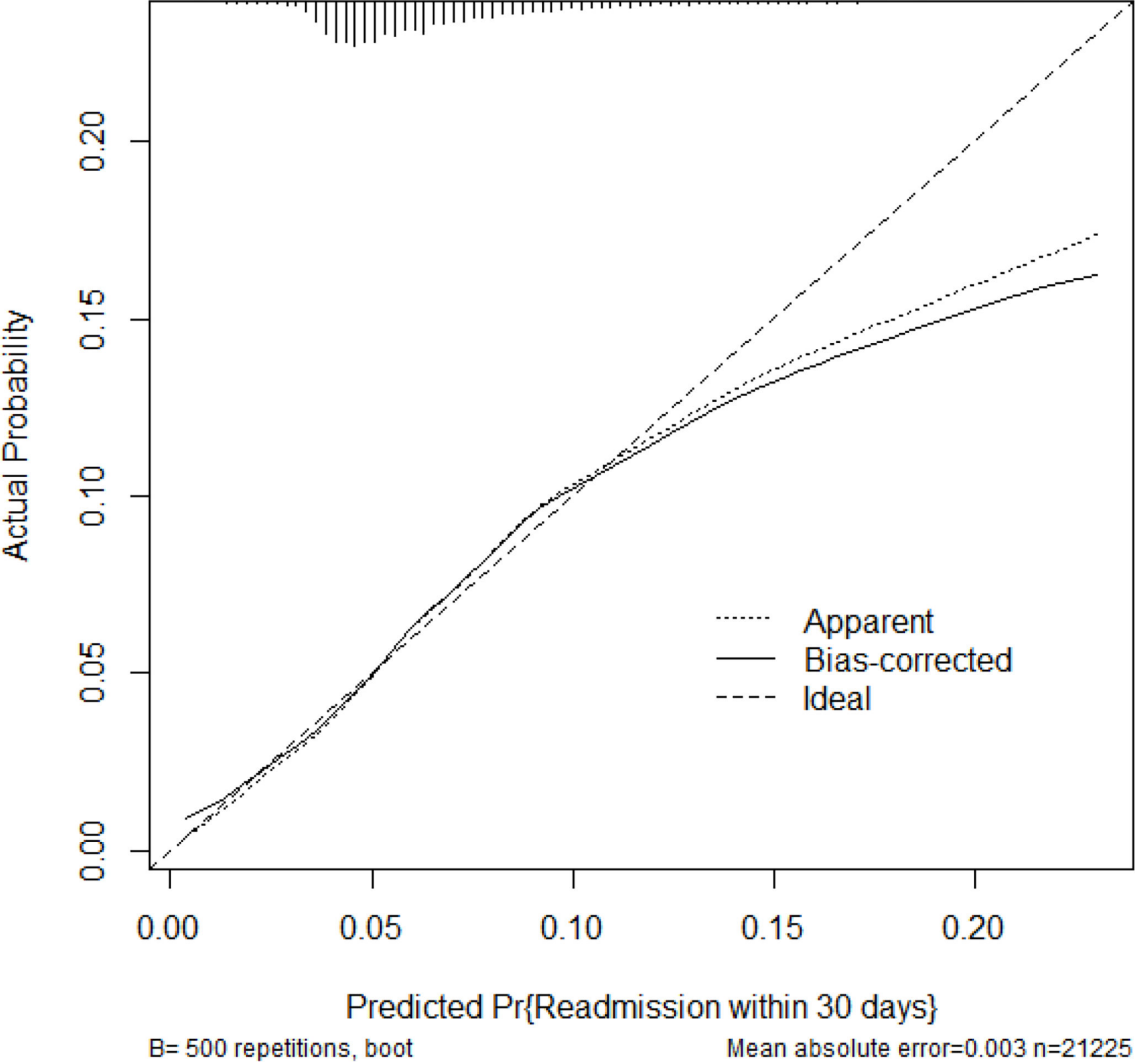
Calibration of readmission prediction.

Completion of preoperative education was not significantly associated with 30-day readmission (Table III and Table IV). The age-education interaction was also non-significant. Readmission risk was primarily associated with patient-level characteristics: older age, higher frailty category, and greater comorbidity strongly increased risk. In contrast, procedure type (THA vs TKA) and early mobilization did not show measurable associations. The model’s calibration curve and discrimination statistics are presented in Figures 6 to 8; discriminative performance was modest with an area under the curve (AUC) of 0.62.

**Table III.** Empirical probability of readmission within 30 days by trust.

Trust	Empirical probability of readmission within 30 days
Blackpool	0.07
Countess of Chester	0.04
East Lancs	0.04
Euxton Hall	0.05
Fairfield Independent	0.05
Fulwood Hall	0.03
Lancs Teaching	0.06
Liverpool University Hospitals	0.08
Manchester FT	0.08
Mersey & W Lancs	0.08
Mid Cheshire	0.06
Northern Care Alliance	0.11
Renacres	0.04
Spire Cheshire	0.04
Spire Liverpool	0.05
Warrington & Halton	0.07
WWL	0.04

WWL, Wrightington, Wigan and Leigh Teaching Hospitals NHS Foundation Trust.

**Table IV.** χ² statistics of readmission model with effect sizes and a 95% CI.

Factor	*χ^2^*	df	p-value
HipKnee	2.0	1	0.200
Age	131	4	< 0.0001
Non-linear	15	2	0.0007
Age, preop education interaction*	5.3	2	0.070
PreopEducation	7.2	3	0.070
Sex	6.4	1	0.010
Walking	0.76	1	0.400
FrailtyRisk	29	2	< 0.0001
Charlson_g	29	2	< 0.0001

* Sub analysis within age- preop education interaction.

### Secondary outcome: early mobilization

**Fig. 9:**
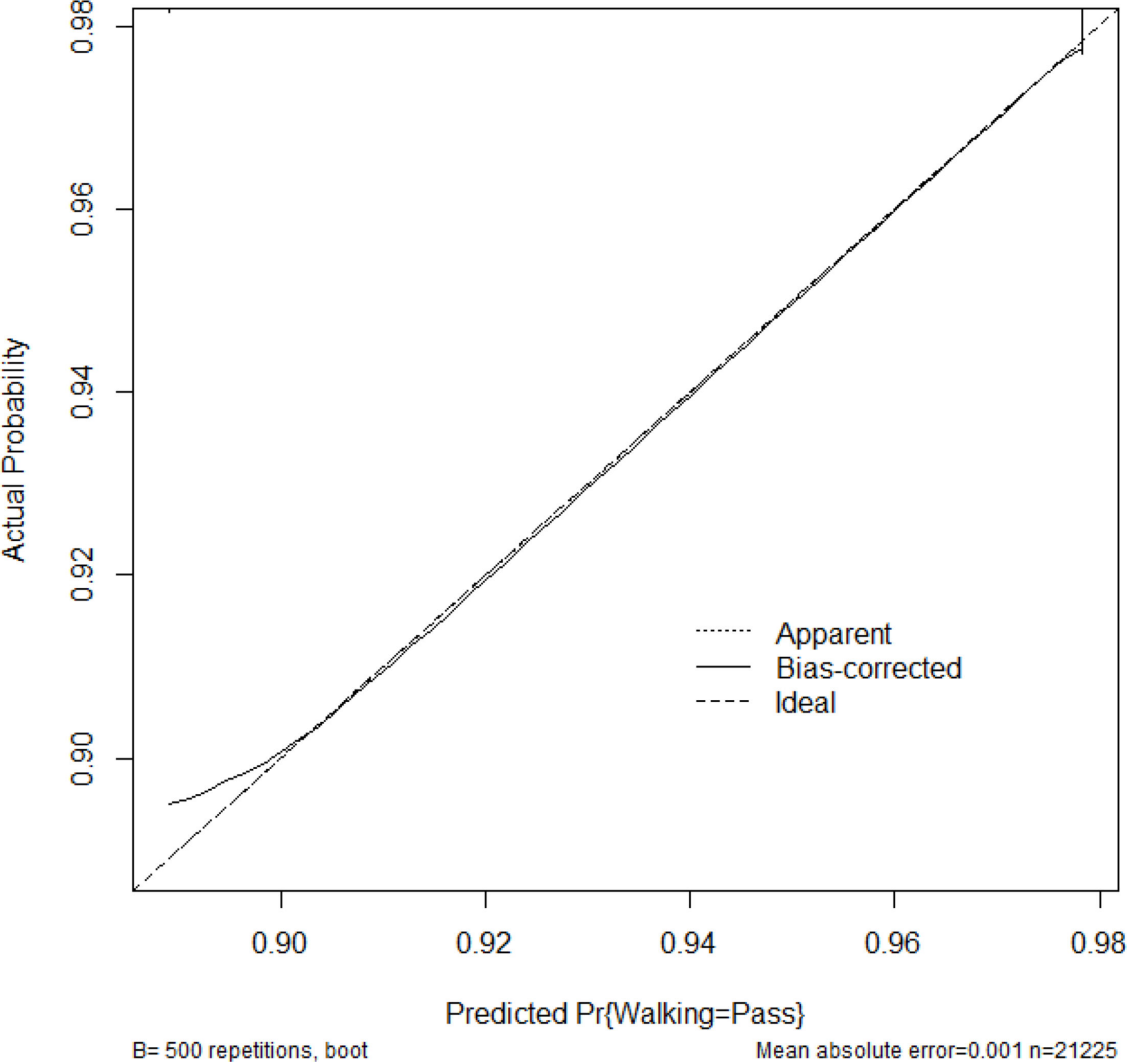
Calibration of the early mobilization model.

Non-completion of preoperative education was associated with reduced odds of early mobilization: *χ^2^*(1) = 6.2, p = 0.010; odds ratio 0.18 (95% CI 0.045 to 0.70). Mobilization performance was generally high across centres; however, trust-level pass rates ranged from 87.9% to 100% (Table V), and the model’s modest discrimination (AUC 0.60; Figure 9) suggests that organizational practice, staffing, and local pathway delivery exert greater influence than preoperative education alone.

**Table V.** Empirical probability of walking pass by Trust.

Trust	Empirical probability of walking pass
Blackpool	0.996
Countess of Chester	0.997
East Lancs	0.995
Euxton Hall	1.000
Fairfield Independent	0.999
Fulwood Hall	1.000
Lancs Teaching	0.909
Liverpool University Hospitals	0.987
Manchester FT	0.997
Mersey & W Lancs	0.998
Mid Cheshire	0.944
Northern Care Alliance	0.879
Renacres	1.000
Spire Cheshire	0.975
Spire Liverpool	0.979
Warrington & Halton	0.988
WWL	0.946

WWL, Wrightington, Wigan and Leigh Teaching Hospitals NHS Foundation Trust.

### Summary of key findings

Preoperative education was associated with reduced LOS, particularly among younger and lower-risk patients. No associations were observed between education and 30-day readmission, but education was associated with early mobilization. Frailty, comorbidity and age were consistently associated with poorer outcomes, particularly prolonged LOS and higher risk of 30-day readmission. Together, these findings highlight the importance of targeted approaches for higher-risk groups.

## Discussion

This multicentre analysis found that completion of preoperative education was associated with a modest reduction in postoperative LOS among patients undergoing hip and knee arthroplasty, with the greatest benefit observed in younger age groups. No association was observed with 30-day readmission, but there was an association with early mobilization. Increasing age, comorbidity burden, and frailty were consistently associated with longer LOS and higher risk of readmission. These findings support the view that patient-level characteristics remain central drivers of perioperative recovery, and that the effects of education are not uniformly distributed across the patient population.

The positive association between education and reduced LOS aligns with systematic reviews demonstrating small but measurable gains in postoperative recovery for patients attending preoperative information sessions.^21,22^ However, much of the existing literature has been derived from single centres delivering standardized curricula.^21,22^ Our findings extend this evidence by evaluating delivery across 17 hospitals in routine NHS practice, with variation in content, format, and timing. The observation that benefits appear concentrated in younger patients may reflect that education is more effective in those with greater cognitive capacity, health literacy, or confidence to engage in self-directed recovery, consistent with broader perioperative behavioural frameworks.^23,24^ Conversely, the attenuated effect in older adults may reflect cognitive burden, reduced retention of complex information, or competing priorities such as comorbid illness.

The absence of association between education and 30-day readmission mirrors evidence suggesting that readmissions are more tightly linked to postoperative complications, comorbidity, and health system delivery rather than preoperative preparation.^25,26^ This is further reinforced by the modest discrimination of the readmission model, which highlights the challenge of risk-stratifying unscheduled returns using routinely collected data. A similar pattern is seen with early mobilization. Although education may theoretically prime patients for activity, mobilization performance is typically determined by availability of therapists and nursing support, weekend compared with weekday operating lists, and local enhanced recovery after surgery (ERAS) culture.^3,21,22^ The present study therefore suggests that patient-facing interventions must be integrated with organizational processes if they are to translate into functional milestones.

Frailty is increasingly recognized as a key determinant of postoperative outcomes in arthroplasty and has been associated with complications, prolonged hospitalization, and greater resource use.^11-17^ The present findings are consistent with this literature, with increasing frailty associated with longer length of stay and higher risk of 30-day readmission after adjustment for demographic and clinical factors. These results reinforce the importance of recognizing frailty as a core perioperative risk factor in elective arthroplasty, and support the need for perioperative pathways that accommodate patients with greater physiological vulnerability.^4,8^ This study did not formally examine frailty-specific predictors of education completion or early mobilization, which may represent an important focus for future work.

These findings support continued use of preoperative education as part of elective arthroplasty pathways, particularly for younger and lower-risk patients who appear most likely to benefit. However, the lack of association with 30-day readmission and limited association with early mobilization suggests that education alone is insufficient to meaningfully alter recovery trajectories for the majority of patients. Stratified perioperative pathways may therefore be justified, with targeted resources directed towards patients with greater physiological or functional vulnerability. Integrating education within broader multimodal perioperative strategies, such as exercise therapy, comorbidity optimization, and psychosocial support, may maximize benefit for older and frailer individuals. There is also scope to improve translation of education into inpatient behaviour. Aligning patient expectations with postoperative ward practices, reinforcing messages through therapy and nursing teams, and adopting consistent mobilization protocols may ensure that patients are better positioned to act on preoperative learning. Programme standardization across trusts, supported by quality assurance frameworks, could also reduce unwarranted variation in delivery.

Strengths of this study include a large multicentre cohort, delivery under routine clinical conditions and adjustment for key confounders, including frailty. The dataset reflects routine arthroplasty activity across a diverse group of NHS and independent hospitals, increasing generalizability. The use of advanced statistical analysis, including restricted cubic splines to model non-linear effects, cluster-robust estimates, and internal validation via bootstrapping, supports the robustness of the findings.

Nevertheless, limitations exist. Retrospective analysis precludes causal inference, and the use of routine data restricts the variables available for adjustment. Education content and delivery mode were not standardized or directly measured, potentially diluting observed effects. Early mobilization was recorded as a binary measure, limiting insight into dose-response or clinical nuance. Additional factors known to influence LOS and readmission, including postoperative complications, pain burden, discharge destination, and social support, were unavailable. These constraints likely contribute to the modest explanatory power of regression models.

In conclusion, completion of preoperative education was associated with reduced length of stay, particularly among younger and lower-risk patients. In contrast, frailty, comorbidity, and age remained the strongest predictors of prolonged stay and early readmission. These results indicate that education alone is insufficient to drive substantial improvements in perioperative outcomes across the arthroplasty population. Incorporating education into integrated multidisciplinary pathways and tailoring support to frailer patients may yield greater benefit. Targeted prehabilitation and ongoing evaluation of personalized delivery approaches may therefore be important considerations for elective pathway optimization, particularly as surgical volumes rise within an increasingly complex patient population.

**Take home message**

- Preoperative education is associated with a modest reduction in length of stay following hip and knee arthroplasty, with the greatest benefit observed in younger and lower-risk patients.

- It does not appear to influence 30-day readmission and has a limited impact on early mobilization. These findings suggest that education should be integrated within broader, targeted perioperative pathways, particularly for older and frailer patients.

## Data Availability

The data that support the findings for this study are available to other researchers from the corresponding author upon reasonable request.
